# Complete Chloroplast Genomes of *Acanthochlamys bracteata* (China) and *Xerophyta* (Africa) (Velloziaceae): Comparative Genomics and Phylogenomic Placement

**DOI:** 10.3389/fpls.2021.691833

**Published:** 2021-06-14

**Authors:** Vincent Okelo Wanga, Xiang Dong, Millicent Akinyi Oulo, Elijah Mbandi Mkala, Jia-Xin Yang, Guy Eric Onjalalaina, Moses Kirega Gichua, Paul Muigai Kirika, Robert Wahiti Gituru, Guang-Wan Hu, Qing-Feng Wang

**Affiliations:** ^1^CAS Key Laboratory of Plant Germplasm Enhancement and Specialty Agriculture, Wuhan Botanical Garden, Chinese Academy of Sciences, Wuhan, China; ^2^University of Chinese Academy of Sciences, Beijing, China; ^3^Sino-Africa Joint Research Center, Chinese Academy of Sciences, Wuhan, China; ^4^Botany Department, Jomo Kenyatta University of Agriculture and Technology, Nairobi, Kenya; ^5^East African Herbarium, National Museums of Kenya, Nairobi, Kenya

**Keywords:** *Acanthochlamys bracteata*, *Xerophyta*, chloroplast genome, comparative genomics, repeat analysis, SSRs, phylogeny

## Abstract

*Acanthochlamys* P.C. Kao is a Chinese endemic monotypic genus, whereas *Xerophyta* **Juss**. is a genus endemic to Africa mainland, Arabian Peninsula and Madagascar with ca.70 species. In this recent study, the complete chloroplast genome of *Acanthochlamys bracteata* was sequenced and its genome structure compared with two African *Xerophyta* species (*Xerophyta spekei* and *Xerophyta viscosa*) present in the NCBI database. The genomes showed a quadripartite structure with their sizes ranging from 153,843 bp to 155,498 bp, having large single-copy (LSC) and small single-copy (SSC) regions divided by a pair of inverted repeats (IR regions). The total number of genes found in *A. bracteata*, *X. spekei* and *X. viscosa* cp genomes are 129, 130, and 132, respectively. About 50, 29, 28 palindromic, forward and reverse repeats and 90, 59, 53 simple sequence repeats (SSRs) were found in the *A. bracteata*, *X. spekei*, and *X. viscosa* cp genome, respectively. Nucleotide diversity analysis in all species was 0.03501, Ka/Ks ratio average score was calculated to be 0.26, and intergeneric K2P value within the Order Pandanales was averaged to be 0.0831. Genomic characterization was undertaken by comparing the genomes of the three species of Velloziaceae and it revealed that the coding regions were more conserved than the non-coding regions. However, key variations were noted mostly at the junctions of IRs/SSC regions. Phylogenetic analysis suggests that *A. bracteata* species has a closer genetic relationship to the genus *Xerophyta*. The present study reveals the complete chloroplast genome of *A. bracteata* and gives a genomic comparative analysis with the African species of *Xerophyta*. Thus, can be useful in developing DNA markers for use in the study of genetic variabilities and evolutionary studies in Velloziaceae.

## Introduction

Velloziaceae is a monocotyledonous family of flowering plants consisting of five genera and c. 250 species (Mello-Silva et al., 2011; Behnke et al., 2013). It is classified under the small but morphologically diverse order Pandanales together with Cyclanthaceae, Pandanaceae, Stemonaceae, and Triuridaceae (Angiosperm Phylogeny Group, 2009; Chase et al., 2016). Basing on its generic limits and distributional patterns, it is one of the most interesting plant families that occur in Africa mainland, Madagascar, Arabian Peninsula, and South America (Porembski and Barthlott, 2000; Ibisch et al., 2001; Alves and Kolbek, 2010). Three genera occur in South America, of which two are endemic to Brazil (*Barbacenia* Vand., *Vellozia* Vand.) and the third occurs in the Andean region (*Barbaceniopsis* L.B.Sm.). A fourth genus, *Xerophyta* Juss., grows in tropical Africa, Arabian Peninsula and Madagascar (Gardens, 2016), and the fifth genus, *Acanthochlamys* P.C. Kao, is endemic to China, native to Tibet and Sichuan (Ibisch et al., 2001; Mello-Silva et al., 2011; Bao-Chun, 2017). Most species of Velloziaceae occur in the tropical regions of South America, and c.70 species occur in the Old world (Behnke et al., 2013). The plant family mainly consists of shrubs and herbs having stems with persistent leaf-sheaths and fibrous root structure (Beentje, 1994). It is the largest lineage of resurrection plants among angiosperms with its species having varying degrees of desiccation tolerance (Alcantara et al., 2015). This is because its species display different strategies to desiccation, with some of species being able to completely avoid desiccation (Alcantara et al., 2015).

The family is one of the classical examples of “Taxonomic nightmares” among plants, due to its floral similarities and the huge variabilities in the morphological features in terms of leaf form, size and life forms among others. Despite the unquestionable uniqueness of the family, there is still serious lack of phylogeographic synthesis about its species. This is because the vast majority of the available studies lack a phylogenetic perspective, and the information generated has been regarded as having little relevance for historical biogeography of both the New World and the Paleotropical species of Velloziaceae. In addition, only few species within the family have had their whole chloroplast genome sequenced including *Xerophyta viscosa* (Farrant et al., 2015), *Xerophyta spekei* (Wanga et al., 2020), and reported through a short communication showing their length and gene contents of their cp genomes. However, a comprehensive comparative analysis of these chloroplast genomes is still lacking.

*Xerophyta* Juss. is a genus that consists of small to large perennial herbs and shrubs, naturally occurring in Africa, Madagascar, and the Arabian Peninsula. Most species of this genus have evolved an adaptation to lose their chlorophyll and terminate the process of photosynthesis during periods of extreme drought hence are extremely desiccation tolerant plants (Tuba et al., 1996). Hence, it has been used in the experimental studies on desiccation tolerance (Deeba and Pandey, 2017). In the same vain, *Acanthochlamys bracteata* P.C. Kao is a dwarf perennial herb found in the grassland nearby bushland of xerophytic valley of China (Deeba and Pandey, 2017). This species was previously classified under the monotypic family Acanthochlamydaceae (Deeba and Pandey, 2017), however, basing on *TrnL* and *rbcL* gene*s* sequence data, it was transferred into the family Velloziaceae (Salatino et al., 2001; Mello-Silva et al., 2011). Additionally, morphological shared characters which are similar in form, structure, and origin, mostly persistent leaves, nucellus, tripartite stem cortex, and phloem tube among others, supported its inclusion into Velloziaceae (Mello-Silva et al., 2011). Morphology, pollen structures and biochemistry have played an important role in the grouping of plants into different taxa (Asaf et al., 2019). However, more emphasis has to be placed on molecular systematics to help understand the morphologically and biochemically similar plants through the genome-wide analysis of their chloroplast.

Systematics and phylogeny, since its inception, has boosted classification and understanding of the evolutionary relationships among plants through genomic analysis (Li et al., 2018; Khan et al., 2019). Furthermore, breeding of drought tolerant crops is key to curb the climate change effects and the growing human population (Dai, 2011; Farrant et al., 2015). Chloroplasts are not only useful in photosynthesis, but also a major genetic system together with the nucleus and the mitochondria (De Las Rivas et al., 2002; Daniell et al., 2016; Moon, 2018; Chen et al., 2019; Khan et al., 2019; Li et al., 2019; Zhou et al., 2019). Due to its highly conserved nature, slow rate of nucleotide substitution and its maternal heredity, Chloroplast DNA (cpDNA) has been widely used in genomics to study plant phylogeny thus an important and informative source for taxonomic and phylogenetic studies (Palmer, 1987; Sale et al., 1993; Lee et al., 2014; Li et al., 2018; Moon, 2018; Wang et al., 2018; Konhar et al., 2019; Li et al., 2019; Liu et al., 2019; Zhang et al., 2019; Oulo et al., 2020). The Plastome is circular, having a quadripartite structure and varies from 120kb to 170 kb, having small single copy (SSC) and Large single copy (LSC) regions, divided by two inverted repeats (IRa and IRb) (Zhao et al., 2015; Wang et al., 2018; Zhou et al., 2019). Plastome phylogenomics has led to tremendous advancements in NGS (next-generation sequencing) technologies hence genome sequencing is currently easier, faster and cheaper (Daniell et al., 2016; Li et al., 2017; Konhar et al., 2019; Khan et al., 2019; Liu et al., 2019). However, despite these advancements in sequencing technologies, there are still few plants that have had their chloroplast genome sequenced (Khan et al., 2019). Additionally, regardless of the uniqueness of Velloziaceae, there is still paucity of information available on the whole chloroplast genomes comparison. This present study reveals the sequenced chloroplast genome of *A. bracteata* and a performed phylogenetic analysis to validate its placement; together with *X. spekei* (MN663122) and *X. viscosa* (NC_043880) from the NCBI database. Additionally, we briefly discuss the morphological comparison between *Xerophyta* and *Acanthochlamys*. This will help in understanding the species in the family and also provide genetic resources for further analyses on the taxonomy and phylogeny of the Velloziaceae.

## Materials and Methods

### DNA Extraction and Sequencing

The fresh green leaves of *A. bracteata* were collected from Luhuo Sichuan at an altitude of 3045 m, China. They were sampled and immediately dried using silica gel in plastic bags (Chase and Hills, 1991). The voucher specimens were stored in the herbarium at Wuhan Botanical Garden, CAS (HIB) (China) with the voucher number DX-0006. 0.5g of the silica dried leaves was used for the DNA extraction using modified cetyltrimethylammonium bromide (CTAB) protocol (Doyle, 1991). Sequencing was done using illumina paired end technology platform at the Novogen Company in Beijing, China.

### Genome Assembly and Annotation

After filtering the low-quality data and adaptors, the obtained clean data was assembled using Get Organelle version 1.7.4 software (Jin et al., 2020b), and then manually corrected. Gene annotation was done using Plastid Genome Annotator (PGA) (Qu et al., 2019) using the plastome of *X. spekei* as the reference genome. Geneious prime and GeSeq online tool^1^ (Tillich et al., 2017), was used to manually edit and correct annotations. The circular chloroplast genome map was drawn using the Organelle Genome DRAW (OGDRAW) software (Greiner et al., 2019). The divergence of *A. bracteata*, *X. spekei* and *X. viscosa* species genomes was determined using mVISTA (Frazer et al., 2004) in the glocal alignment algorithm (shuffle-LAGAN mode) and using *A. bracteata* as the reference genome.

### Analysis of Repeats and Codon Usage

Long repeat sequences (forward, reverse, complimentary, and palindromic) in the genome sequence were identified using REPuter online program (Kurtz et al., 2001). Locations and sizes of the repeat sequence were visualized with a minimal standard of: (1) minimum repeat size of 30bp, (2) a hamming distance of 3, (3) 90% or greater identity. Tandem repeats in the 3 species of Velloziaceae; *X. viscosa*, *X. spekei*, and *A. bracteata* cp genomes were identified using the Tandem repeat finder (Benson, 1999) with inbuilt alignment parameters. Simple Sequence Repeats (SSRs) analysis was done using the Perl script Microsatellite (MISA)^2^ (Thiel et al., 2003), considering a nucleotide size of 1 to 6 base pairs and a threshold of 10, 5, 5, 3, and 3 for mono-, di-, tri-, tetra-, penta-, and hexa-nucleotides, respectively. The codon bias (RSCU) in the three species was conducted using MEGA7 software (Kumar et al., 2016).

### Nucleotide Diversity and Substitution Rate Analysis

To assess the nucleotide diversity (*Pi*) in the complete Plastome of the three species, *A. bracteata* was compared with the species *X. spekei* and *X. viscosa*. The complete chloroplast genome (cpDNA) sequences were aligned using MAFFT in-built in phylosuite (Zhang et al., 2018). A sliding window analysis of window length of 600 bp and step size of 200 bp, was used in the DnaSP to estimate the nucleotide diversity values of each gene (Rozas et al., 2017). Protein-coding genes of *A. bracteata*, *X. spekei*, and *X. viscosa* were extracted using Phylosuite, aligned using MAFFT and Ka/Ks rates for each gene estimated using the Ka/Ks calculator (Zhang et al., 2006). Selection pressure within the shared genes of the eleven species of the order Pandanales was evaluated using PAML v4.7 (Yang, 2007), executed in the EasyCodeML software (Gao F. et al., 2019). The dN/dS ratio of the species of the order Pandanales (*Pandanus tectorius, Carludovica palmata, Stemona tuberosa, Stemona mairei, Stemona japonica, Croomia pauciflora, Croomia heterosepala, Croomia japonica, X. spekei, X. viscosa*, and *A. bracteata*) was also calculated based on four site specific models (M0 vs. M3, M1a vs. M2a, M7 vs. M8 and M8a vs. M8) with likelihood ratio test (LRT) threshold of *p* < 0.05 to show highly variable sites in the genome. The protein-coding genes were aligned in correspondence to their amino acids and selection pressures on the genes analyzed using both ω and LRTs values. We estimated the interspecific genetic distance with MEGA X using Kimura two-parameter (K2P) model^3^ (Kumar et al., 2018).

### Phylogenetic Analysis

To understand the phylogenetic relationship of *A. bracteata* P.C. Kao with other species of the Order Pandanales, maximum likelihood (ML) and Bayesian inference (BI) trees were reconstructed. We generated 59 individual plastid gene files representing the shared protein-coding genes. Other representatives of the Velloziaceae (*Vellozia sp*., *Xerophyta elegans*, *Barbacenia involucrata*, *Barbaceniopsis castillonii*) were sampled from the gene bank based on a previous study (Soto Gomez et al., 2020). The 59 shared protein-coding genes of 55 species representatives from orders; Pandanales, Dioscoreales, and Liliales, were used to reconstruct the phylogeny using *Elaeis guineensis* as an outgroup based on previous study (Liu et al., 2012) (Supplementary Table 6). All the 55 species were subjected to MAFFT alignment, and the phylogenetic relationships estimated using the ML and BI analyses done using the IQ-Tree and MrBayes, respectively, integrated in Phylosuite (Zhang et al., 2018) (Supplementary Table 6). Model Finder (Kalyaanamoorthy et al., 2017) was used to find the best model using Bayesian Information Criterion (BIC). The model of best-fit for Bayesian analysis was GTR + F + I + G4, while that of IQ-tree was GTR + F + R3. The models GTR + I + G4 + F and GTR + F + R3 was run for 1000 replicates using ultrafast bootstraps.

## Results and Discussion

### Complete Chloroplast Genomes

**FIGURE 1:**
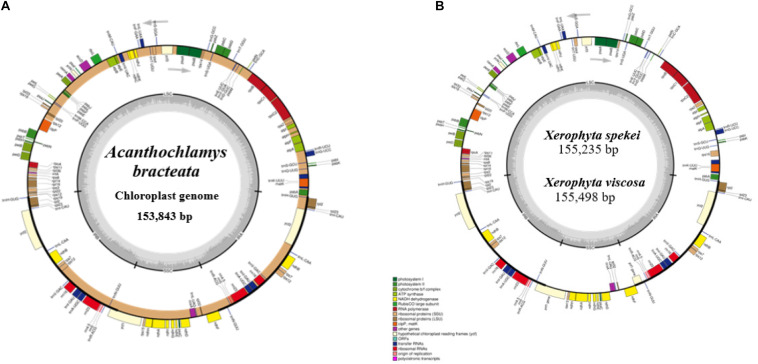
Genome of map of: **(A)** *Acanthochlamys bracteata*; **(B)** *Xerophyta spekei*, and *Xerophyta viscosa*. The genes shown inside and outside the circular maps are transcribed clockwise and anticlockwise directions, respectively. Genes from different functional groups are shown in different colors. The thick dark lines show the extent of the Inverted repeats (IRa and IRb) separating the Large Single-Copy (LSC) and the Small Single-Copy (SSC) regions. The dark gray and the light gray lies embedded inside the circle represent the GC and the AT content, respectively.

The complete chloroplast sequence of *A. bracteata* was deposited in the GenBank database (Accession No. MW727487). All the three species’ whole chloroplast genome; *A. bracteata*, *X. spekei*, and *X. viscosa* exhibited a spherical quadripartite nature (Figure 1), with sizes of *A. bracteata*, *X. spekei*, and *X. viscosa* cp genomes being 153,843 bp, 155,235 bp (Wanga et al., 2020), and 155,498 bp, respectively, similar to most angiosperm Plastomes (Daniell et al., 2016). The cp genomes consist of Inverted repeats (IRs) (IRa and IRb) each with a length ranging from 27,022–27,110 bp within the three species. The Large Single-Copy (LSC) region in the three species showed length ranging from 81,919 to 83,813 bp and Small Single-Copy (SSC) region (17,387–17,880 bp) (Table 1). The LSC and SSC regions are separated by the IRs. Generally, the gene constituent of the chloroplast genome is approximately between 120 and 140 genes that are always actively involved in photosynthesis, transcription and translation processes (Gu et al., 2019). All the genes annotated in the cp genomes of the three species ranged between 129 and 132 genes, including 37–38 tRNAs and 8 rRNAs. The guanine-cytosine (GC) content of the three chloroplast genomes showed no significant difference, however, *A. bracteata* had a slightly lower GC content of 37.4% of the genome. The regions (LSC and SSC) had no considerable differences in the GC content in the three species. However, the IR regions showed a higher GC content of 42.6%. This is due to the presence of the rRNA and tRNA genes which occupy greater area than the protein-coding genes within the inverted repeat regions (Table 2). This phenomenon has also been shown in previous studies (Talat and Wang, 2015; Chen et al., 2016).

**TABLE 1:** Composition of *Acanthochlamys bracteata*, *Xerophyta spekei*, *Xerophyta viscosa* cp genomes with related species of the Order Pandanales.

Species name	GenBank Accession no.	Total Length (bp)	LSC (bp)	SSC (bp)	IR (bp)	PCGs region size (bp)	tRNA size (bp)	rRNA size (bp)	Total GC content (%)	Total No. of PCGs	Total No. of tRNA	Total No. of rRNA	No. of genes
*Acanthochlamys bracteata*	MW727487	153,843	81919	17880	27022	78,987	2,794	9,050	37.4	84	37	8	129
*Xerophyta spekei*	MN663122	155,235	83628	17387	27110	79,077	2,796	9,052	37.6	85	37	8	130
*Xerophyta viscosa*	NC_043880	155,498	83813	17479	27103	78,957	2,870	9,052	37.6	85	38	8	132
*Stemona tuberosa*	MW246829	154,374	82305	17929	27070	80,436	2,877	8,528	37.9	88	38	8	134
*Stemona mairei*	NC_039676	154,307	82254	17889	27082	79,254	2,877	9,060	38.0	87	38	8	134
*Stemona japonica*	NC_039675	154,224	82117	17943	27082	79,284	2,877	9,060	38.0	87	38	8	134
*Croomia pauciflora*	NC_039674	155,261	82427	18346	27244	79,509	2,877	9,052	38.3	87	38	8	134
*Croomia heterosepala*	NC_039673	154,407	81844	18145	27209	79,344	2,859	9,052	38.3	87	38	8	134
*Croomia japonica*	NC_039672	154,672	81981	18271	27210	79,344	2,931	9,052	38.3	87	38	8	134
*Sciaphila densiflora*	KR902497	21,485	−	−	−	9,795	443	4,531	39.9	18	6	4	24
*Carludovica palmate*	NC_026786	158,545	87041	18366	26569	79,197	2,877	9,052	37.7	86	38	8	133
*Pandanus tectorius*	NC_042747	159,362	87445	18509	26704	55,650	2,876	9,052	37.6	82	38	8	125

**TABLE 2:** Features of the LSC, SSC and IR of the Order Pandanales’ Plastomes.

Species name	Large Single-copy (LSC)			Small Single-Copy (SSC)			Inverted Repeat (IR)		
	Total Length (bp)	Total GC (%)	Length in (%)	Total Length (bp)	Total GC (%)	Length in (%)	Total Length (bp)	Total GC (%)	Length in (%)
*Acanthochlamys bracteata*	81919	35.3	53.2	17880	31.3	11.6	27022	42.6	17.6
*Xerophyta spekei*	83628	35.5	53.9	17387	31.8	11.2	27110	42.6	17.5
*Xerophyta viscosa*	83813	35.5	53.9	17479	31.8	11.2	27103	42.6	17.4
*Stemona tuberosa*	82305	36.0	53.3	17929	31.9	11.6	27070	42.7	17.5
*Stemona mairei*	82254	36.2	53.3	17889	32.2	11.6	27082	42.7	17.6
*Stemona japonica*	82117	36.2	53.2	17943	32.1	11.6	27082	42.7	17.6
*Croomia pauciflora*	82427	36.6	53.1	18346	32.5	11.8	27244	42.9	17.5
*Croomia heterosepala*	81844	36.6	53.0	18145	32.3	11.8	27209	42.8	17.6
*Croomia japonica*	81981	36.6	53.0	18271	31.8	11.8	27210	42.5	17.6
*Carludovica palmate*	87041	35.8	54.9	18366	31.3	11.6	26569	42.5	16.8
*Pandanus tectorius*	87445	35.6	54.9	18509	31.9	11.6	26704	42.8	16.8

*LSC, Large Single-Copy; SSC, Small Single-Copy; IR, Inverted repeat.*

The genomes contain protein-coding genes ranging from 84 or 85 in number, transfer RNA genes (tRNA) ranges from 37 or 38 in number, and 8 rRNA genes (Table 1). A significant number of genes occur in the LSC and SSC regions. However, 17 genes are recurrent in the inverted repeat (IRa and IRb) regions. These include six coding genes (*ndhB*, *rpl2*, *rps12*, *rpl23*, *rps7*, *ycf2*); and the non-coding include seven transfer RNA species (*trnA-UGC*, *trnI-CAU*, *trnI-GAU*, *trnH-GUG*, *trnN-GUU*, *trnR-ACG*, and *trnV-GAC*) and four ribosomal RNA species (*rrn4.5*, *rrn5*, *rrn16*, and *rrn23*). *ycf1* gene is a pseudogene which extends through the SSC and IR regions. *rps12* gene is located in both the LSC and the Inverted repeat (IR) regions. 63 protein-coding genes and 21 tRNA genes are found in the LSC region, while the SSC region has 12 protein-coding genes and 1 tRNA; and the IR region contain 7 PCGs and 7tRNA. The protein-coding genes in the *A. bracteata* cp genome include: *rpl2*, *rpl14*, *rpl16*, *rpl20*, *rpl22*, *rpl23*, *rpl32*, *rpl33*, *rpl36, rps2*, *rps3*, *rps4*, *rps7*, *rps8*, *rps11*, *rps12*, *rps14*, *rps15*, *rps18*, *rps19, psaA*, *psaB*, *psaC*, *psaI*, *psaJ, psbA*, *psbB*, *psbC*, *psbD*, *psbE*, *psbF*, *psbH*, *psbI*, *psbJ*, *psbK*, *psbL*, *psbM*, *psbN*, *psbT*, *psbZ, atpA*, *atpB*, *atpE*, *atpF*, *atpH*, and *atpI*, which are largely involved in the functions of self-replication and photosynthesis. These patterns of protein-coding genes were found to be present in all the three species of Velloziaceae. Of the 84 protein-coding genes in *A. bracteata*, eight (*ndhA*, *ndhB*, *rpl2*, *rpoC1*, *atpF*, *petB*, *petD*, and *rpl16*) contained one intron, while *clpP* and *ycf3* each contained two introns (Supplementary Table 1). Introns generally play a critical role in regulating and signaling the expression of genes within the species (Daniell et al., 2016). Most importantly, plastome comparative studies provide key information on the present functional genes, rearrangements and genetic mutations that are key drivers of evolution in relation to their prehistorical origin (Sabater, 2018).

The gene content of the cp genomes of *A. bracteata*, *X. spekei*, and *X. viscosa* are shown in Tables 1, 2. Though, no significant differences were recorded in the number of encoded genes, the type of genes or the content of the GC of the three species, which suggests a focus on the intergenic regions for variations.

### Repeat Analysis

**FIGURE 2:**
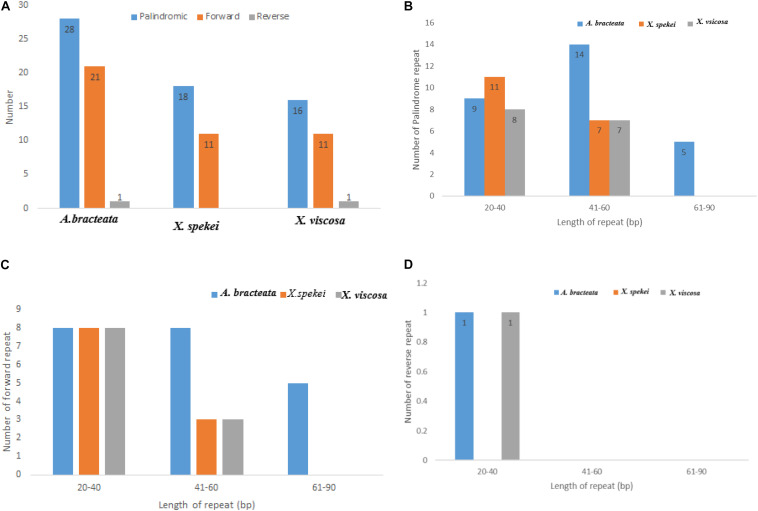
Analysis of the repeat sequences in the whole chloroplast genomes of *A. bracteata*, *X. spekei*, and *X. viscosa*. **(A)** Total abundance of the three repeats; **(B)** Total number of the Palindromic repeats; **(C)** Total number of the forward repeats; **(D)** Total number of the reverse repeats.

Chloroplast repeats are important genetic resources that play a key role in the genome recombination and rearrangement (Lee et al., 2014). They are useful in the study of population genetics and biogeographic studies (Xie et al., 2018). In the current study, repeat analysis revealed that the Plastomes of the three species contained varied number of repeats (i.e., Palindromic, Forward, and Reverse repeats). The repeat analysis of *A. bracteata* revealed palindromic repeats (28), forward repeats (9) and reverse repeats (1). Out of which 9 palindromic, 8 forward and 1 reverse repeat are have a length of between 20 and 40 bp (Figure 2). In *X. spekei* there were only 18 palindromic repeats and 11 forward repeats with no reverse repeats. On the other hand, *X. viscosa* repeat analysis revealed 16 palindromic repeats, 11 forward repeats and 1 reverse repeat. Basing on the type of repeats, *A. bracteata* and *X. viscosa* showed similarity as compared to *X. spekei*. However, in terms of the number and length, there is a variation in the three species.

Microsatellites are small repeating units (1–6 nucleotide) within a genome nucleotide sequence (Shukla et al., 2018). They exhibit polymorphism and are usually dominantly expressed at the species level hence used as DNA markers for the population and evolutionary studies (Vendramin et al., 1999*;* Deguilloux et al., 2004; Piya et al., 2014; Redwan et al., 2015; Gao et al., 2018). In this study, we analyzed the presence, type, and allocation of SSRs in the cp genomes of *A. bracteata, X. spekei*, and *X. viscosa*. Mono-, di-, tri-, tetra-, and hexa-nucleotides types of SSRs were detected in the chloroplast genome of the three species.

**FIGURE 3:**
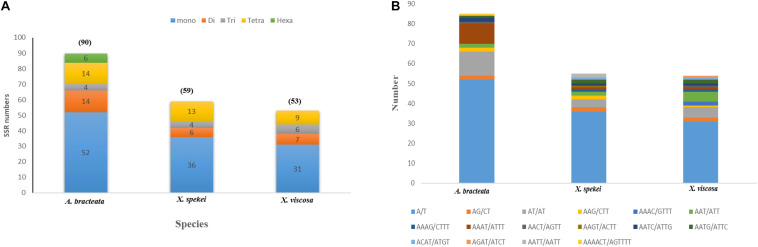
Plastome simple sequence repeats (SSRs) in the three species. **(A)** Total number of different SSR types identified in each species; **(B)** the type and total number of each SSR.

90 SSRs were detected in *A. bracteata* cp genome. Comparably, 59 and 53 microsatellites were revealed in *X. spekei* and *X. viscosa*, respectively (Figure 3A). The mono-nucleotide repeats reported 58.91% of the total SSRs which made them the most abundant type of SSRs within the three species’ cp genomes. Their numbers vary from 52 in *A. bracteata*, 36 in *X. spekei* and 31 in *X. vsicosa*, followed by tetra-nucleotide repeats (17.82%), di-nucleotide repeats (13.37%), tri-nucleotide repeats (6.93%). Penta-nucleotide repeats (2.97%) were the least abundant and were only present in the species *A. bracteata*. The genes within the chloroplast genomes are always highly conserved, however, the microsatellite abundance varies among the species (Gao et al., 2018). A/T mononucleotide repeats were highest in number in all the cp genomes of the three species (Figure 3B). Our findings are similar to other studies that show that A/T repeats were the most abundant (Munyao et al., 2020). However, this varies among species with other studies recording di-nucleotides and tri-nucleotides as most abundant (Wang et al., 2017; Xie et al., 2018). Thus, this shows that SSRs are vital for understanding intrageneric and intergeneric variations within *A. bracteata* and its close relatives in Africa and South American species.

Our results show that SSRs within these chloroplast genomes are mostly comprised of poly-adenine (Poly-A) and poly-thymine (Poly-T) repeats. Hence, they contribute much to the AT abundance of the three species cp genome. The coding sequences also had SSRs mostly composing of the mono-nucleotide A/T which accounts for only 9.9%. This means that SSRs are mostly located in the non-coding regions. This trends have been shown in previous several studies (Rajendrakumar et al., 2007; Gandhi et al., 2010). These SSRs can be used to develop specific markers, which can be key in the study of systematics and evolution of the family.

### Codon Usage

**FIGURE 4:**
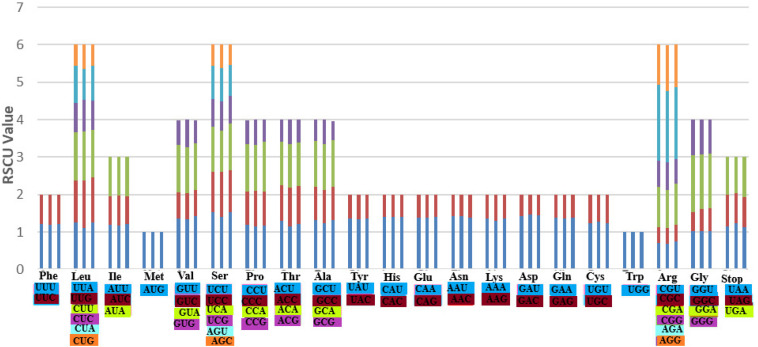
The codon usage bias in all the protein-coding genes of the chloroplast genomes of the three species *A. bracteata*, *X. spekei*, and *X. viscosa*.

Codon usage is an essential feature for gene expression in both eukaryotes and prokaryotes genomes due to its strong correlation to protein and mRNA levels genome-wide (Lyu and Liu, 2020). It is the fact that different organisms vary in their synonymous codons rates of occurrences in their protein-coding sequences, meaning that some codons are rarely used while other codons are frequently used in a particular organism. Based on the protein-coding genes in the three species: *A. bracteata*, *X. spekei*, and *X. viscosa*, 51,281, 51,745, and 51,832 codons, respectively, were identified. Methionine and Tryptophan amino acids are encoded by a single codon. Other amino acids showed obvious codon usage bias. On average, the most abundant amino acids in the three species were leucine (*A. bracteata* 51341; 10.01%, *X. spekei* 5260; 10.17%, *X. viscosa* 4953; 9.56%) whereas the least abundant amino acid was Cysteine (*A. bracteata* 1144; 2.23%, *X. spekei* 1073; 2.07%, *X. viscosa* 1154; 2.23%). The relative synonymous codon usage (RSCU) analysis showed that *A. bracteata* (32 codons), *X. spekei* (33 codons), and *X. viscosa* (33 codons) were >1, indicating a codon bias in the amino acids (Figure 4). Most (28 codons) of these preferred codons in the three species, ended in an A or U. Codon usage bias is a product of selection and mutation factors (Xu et al., 2011; Liu et al., 2018). Hence, the choice of codons within the plastome can be used to show gene expressions and speciation mechanisms in species.

### Nucleotide Diversity (Pi) and Selection Pressure Analysis

**FIGURE 5:**
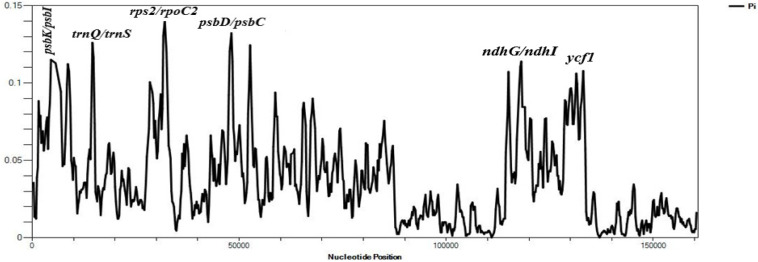
Sliding window analyses of the three whole cp genome sequences using a window length of 600 bp and step size of 200 bp. The nucleotide diversity (*Pi*) value of each window is shown on the Y-axis, and their positions showed on the X-axis.

The average *Pi* value of the three species of Velloziaceae was found to be 0.03501. IR regions showed lower nucleotide diversity indicating that they were quite conserved than the LSC and SSC regions (Figure 5). The nucleotide diversity (*Pi*) showed values ranging from 0.00111 to 0.14000 in the shared protein-coding genes. 20 regions showed values of *Pi* > 0.1000. These results indicated insignificant variations within these genome regions. However, high variations (Pi value > 0.1000) were found in these regions; *psbK-psbI*, *trnQ-UUG*/*trnS-GCU*, *atpA*, *atpF*, *rps2*, *psbD*/*psbC*, *ndhK*/*ndhC*, *atpB*/*rbcL*, *ndhD*, *ndhG*/*ndhI*, *trnA-UGC*, *ycf1*. The Large Single-Copy region (LSC) recorded most of the highly diverse regions.

**FIGURE 6:**
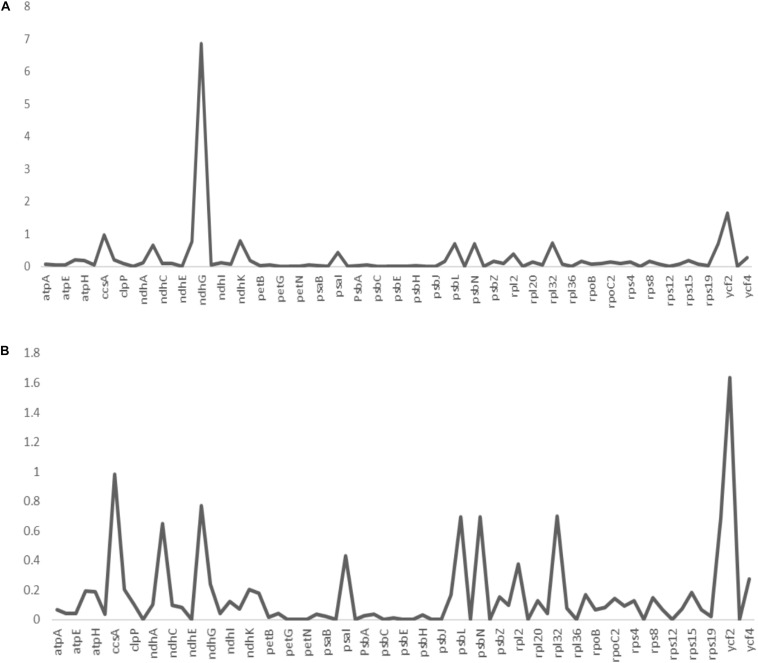
The non-synonymous-to-synonymous substitution (Ka/Ks) ratios of 73 coding genes of **(A)** *Acanthochlamys bracteata* plastome relative to *Xerophyta viscosa* and **(B)** *Acanthochlamys bracteata* plastome relative to *Xerophyta spekei*. Note: Y- axis represents the Ka/Ks values (ratios) while the X- axis represents the protein-coding genes within the chloroplast genomes.

Non-synonymous/Synonymous mutation ratio (Ka/Ks) is comprehensively effective in the detection of selection pressures in proteins or fragments of DNA sequences in plant species (Wang et al., 2010; Gao L. Z. et al., 2019; Xu and Wang, 2021). It is key in analyzing the evolutionary pressures within the genome. Synonymous substitutions are likely to occur in most protein-coding regions compared to non-synonymous substitutions (Rono et al., 2020). The synonymous substitutions leaves the amino acid unchanged as opposed to the non-synonymous substitutions which changes the sequence of the amino acid. In the current study, the estimated Ka/Ks ratios of the 73 protein-coding genes of *A. bracteata*, computed against the close relatives *X. spekei* and *X. viscosa* are shown in the line graphs below (Figure 6). The mean Ka/Ks ration for all the genes was 0.26. For protein-coding genes in the *A. bracteata* plastome, Ka/Ks ratios was majorly between 0 and 1. This suggest that the majority of the genes in the *A. bracteata* plastome were probably under purifying selection. The *ccsA*, *ndhG*, *psbL*, and *ycf2* gene families had higher Ka/Ks ratios compared to most of the rest of the protein-coding genes in the plastome. Two genes (*ycf2* and *ndhG*) showed a Ka/Ks ratio greater than 1 (Supplementary Table 2) in the pairwise comparisons, showing that they may have undergone some evolutionary pressures. Genes with Ka/Ks > 0.5 were *ccsA*, *ndhB*, *ndhF*, *psbL*, *psbN*, *ycf1*, and *ycf2* in the computation against *X. spekei*; whereas in the estimation against *X. viscosa*, the genes included *ccsA*, *ndhB*, *ndhF*, *ndhG*, *ndhK*, *psbL*, *psbN*, *rpl32*, and *ycf2* (Supplementary Table 2). In both estimations of the *A. bracteata*, the least significant Ka/Ks value (0.00) was found mostly in the genes involved in the processes of photosynthesis (*psaC*, *ndhE*, *petG*, *psbJ*, *petL*, *petN*, *psaJ*, *psbC*, *psbE*, *psbF*, *psbI*, *psbM*, and *psbT*), self-replicating genes (*rpl14*, *rpl36*, *rps7*, *rps12*) and hypothetical chloroplast reading frames (*ycf3*) indicating significant purifying selection. Similar results were reported for other cp genomes (Li et al., 2015). These three species occur in more or less similar habitat growing on inselbergs in China and Africa hence a conjecture that functional genes in the chloroplast genomes played a big role in the adaptations in these strenuous environments.

**FIGURE 7:**
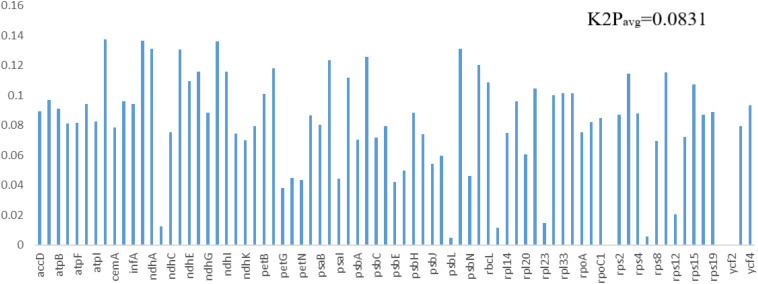
The K2P values estimated for the genes in the cp genomes of the 11 species in the Order Pandanales.

The same protein-coding genes in the order Pandanales were used to detect sites of positive selection within their genomes. Four models (M0 vs. M3, M1a vs. M2a, M7 vs. M8 and M8a vs. M8) were compared in this analysis. Comparative model of M7 vs. M8 was positive in determining the LRT *p* value of >0.05 and the strength of positive selection of the genes. Bayes Emperical Bayes (BEB) and naïve empirical Bayes (NEB) analysis was only shown in model M8. Most genes showed no significant positive selection (p-value > 0.05) except eight genes with high posterior probabilities found in the BEB test (*rps16*, *atpF*, *atpH*, *rpoC2*, *psaA*,*atpB*, *rbcL*, *accD*) and NEB test (*rps16*, *atpI*, *rpoC2*, *psbZ*, *rps14*, *ndhC*, *rbcL*, *accD*) (Supplementary Tables 3, 4). Most of these genes that contained highly positively selected sites were mostly related to functions of photosynthesis and self-replication. Studies have linked these to codon sites with high posterior sites to be under positive selection pressure (Xie et al., 2018). Using K2P model in MEGA, the average interspecific genetic distance in the 77 PCGs for the 11 species in the Order Pandanales was estimated. On average, the K2P interspecific genetic distance was calculated to be 0.0831 (Figure 7 and Supplementary Table 5). The least K2P values were detected in *psbL* gene (0.0050) and the highest was in the gene *ccsA* (0.1374).

**FIGURE 8:**
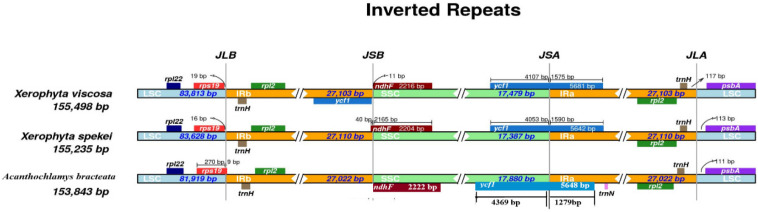
Junction sites comparison of the chloroplast genomes of the three species. JLB: junction line between LSC and IRb; JSB: junction line between IRb and SSC; JSA: junction line between SSC and IRa; JLA: junction line between IRa and LSC.

We compared the border structure of the three cp genomes in detail to identify the IR expansion or contraction (Figure 8). IR regions contained *rpl2* and *trnH* genes. The location of the *ndhF* gene was in the SSC region, however, in the *X. spekei* it was located at the border junction of IRb and SSC region. The *ycf1* gene is a pseudogene found at the junction of IRa and SSC region. In *X. spekei* and *X. viscosa*, the *rps19* was located in the LSC region together with *rpl22* gene, however, in *A. bracteata* the *rps19* extended into the IRb with a 9 bp. There was a notable difference in the inverted repeat regions of the three species. The distance between *psbA* and the JLA junction varies from 111 bp to 117 bp. Inverted repeat regions in land plants’ cp genomes vary greatly (Khayi et al., 2020). However, studies show that they are the most conserved regions of the chloroplast genome (Asaf et al., 2020). Contraction and expansion of the IR regions leads to the size differences in the Plastomes (Mo et al., 2020). IRs are thought to stabilize the plastome with studies showing that Plastomes that lack one or all IRs are less stable in terms of their genome arrangements than the species genome that have the IRs (Jin et al., 2020a, b).

**FIGURE 9:**
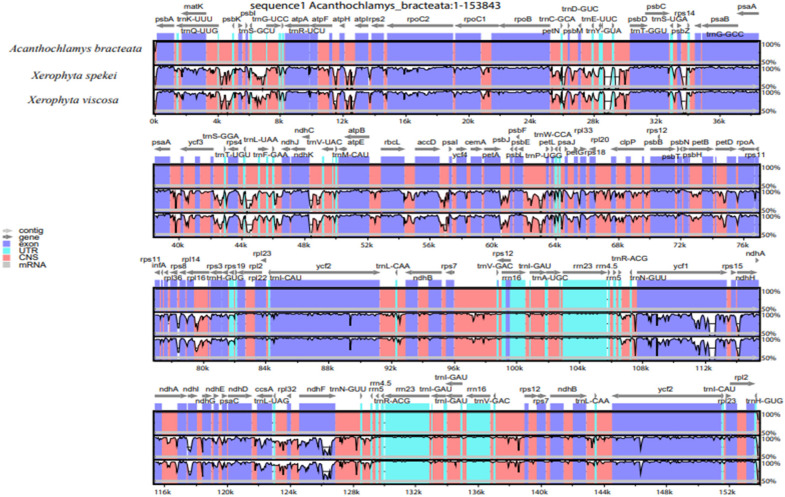
DNA sequence comparison of the three chloroplast genomes using mVISTA program. Above gray lines show the gene orientation and the position in the LSC, SSC, and IRs regions. The vertical scale indicates the percentage of identity of 70%.

To understand structural characteristics of the cp genomes of the three species of Velloziaceae, whole sequence alignment was conducted using the annotation of *A. bracteata* as a reference (Figure 9). These three species all belong to the family Velloziaceae. The gene number, order and orientation were relatively conserved, although some highly divergent regions were found. The results show that all the three species cp genomes were 70% similar. High genetic inconsistency, however, occurred in the single-copy (LSC and SSC) regions compared to the IR regions. Similarly, non-coding regions also had higher gene variations than in the coding regions. The LSC and SSC regions are more divergent than the two IR regions. In addition, within the LSC and the SSC regions, the non-coding regions are more divergent than the coding regions. This kind of phenomenon has been shown in other studies (Wang et al., 2018). The most highly divergent regions include *psbI-trnS*(GCU), *trnS(GCU)-trnG(UCC), ycf3-trnT(UGU), matK*, *psbK*, *ycf2*, *ndhF*, *rpl32*, *ycf1*, *ndhE*, *ndhD*, *ndhA*, *rps4*, *trnH-psbA*, *trnG-psaA, atpB-rbcL*, and *ndhF-rpl23*. The IR regions were highly conserved in terms of gene order and abundance. However, at the border of the IR and single-copy regions there were notable significant differences. Hence, Velloziaceae Plastomes were quite well conserved, with few variations detected (Figure 9). Variations in the size of the genome, expansion and contraction of the IR junctions were the major differences in the 3 cp genomes structure. DNA barcodes are sections of DNA sequences with a high mutation rate that can be useful to identify a species in a given taxonomic group (Rousseau-Gueutin et al., 2015; Xu et al., 2015; Zhou et al., 2016). These major regions of variations can be important markers for barcoding and studies on the evolution within the species of Velloziaceae.

### Phylogenetic Analysis

**FIGURE 10:**
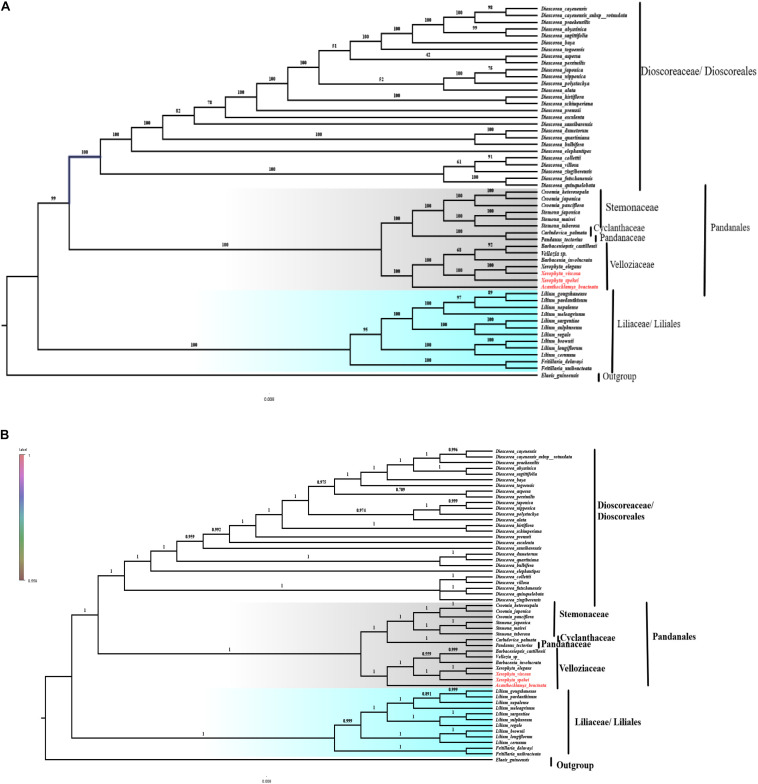
Phylogenetic relationship of the three species of Velloziaceae based on 59 single-copy genes shared by all cp genomes. **(A)** The Maximum likelihood (ML) tree with bootstrap value on the branches; **(B)** Bayesian inference (BI) tree with posterior probabilities values on the branches.

Plastomes are important for explaining intra-and interspecific evolutionary histories with recent studies showing significant power in phylogenetic, evolution, and molecular systematic studies (Asaf et al., 2018). The cp genomes that have sufficient variable sites have shown to be useful in solving the phylogenetic relationships (Ma et al., 2014; Carbonell-Caballero et al., 2015). The phylogeny of the Velloziaceae was reconstructed using 59 shared protein-coding genes from the orders; Pandanales, Liliales and Dioscoreales, to evaluate the position of *Acanthochlamys* and the *Xerophyta* species. The three selected orders (Liliales, Pandanales, and Dioscoreales) were clustered into three different clades. In the reconstructed phylogeny (Figure 10), all the families for the order Pandanales were Cyclanthaceae, Pandanaceae, Stemonaceae, and Velloziaceae with exception of Triuridaceae which has lost most of its genes over time. The families in the Order Pandanales form a monophyletic group at this current taxon sampling analysis. *Acanthochlamys bracteata* was the early diverged in the family Velloziaceae based on this analysis because it was sister to the rest of the Velloziaceae species. *Xerophyta spekei* was sister to the clade that consisted of *Xerophyta elegans* and *Xerophyta viscosa* with a high support (100). *Barbacenia involucrata* L.B.Sm. was sister to the group [*Vellozia sp.* + *Barbaceniopsis castillonii* (Hauman) Ibisch] which was different to the previous analysis that used three genes (Soto Gomez et al., 2020). However, the tree topology of the Velloziaceae clade was similar to the same previous study that performed the analysis using a 12-gene mitochondrial data set. Pandanaceae and Cyclanthaceae clustered together and this clade was sister the clade formed by Velloziaceae and Stemonaceae. The phylogenetic relationship of the taxa in this study was consistent to the previous study that combined *18S rDNA* (mitochondrial), Nuclear *atpA*, *matR*, and *nad1b-c* intron dataset (Mennes et al., 2013) and showed that Velloziaceae was sister to the other families in the order Pandanales, whereas, Stemonaceae was related to the clade (Pandanaceae + Cyclanthaceae; which are sisters). However, in our study we excluded family Triuridaceae from the analysis due to the huge number of genes lost from its cp genome. This phylogenetic tree topology therefore showed a close relationship between the taxa. Furthermore, the three species of Velloziaceae analyzed, *A. bracteata* and the two *Xerophyta* species (*X. spekei*, and *X. viscosa*) clustered together in the same clade, hence revealing a closer relationship of these species. This is similar to an early phylogenetic inference of the family Velloziaceae using the Chloroplast *trnL-F* sequence which supported a close relationship between *Acanthochlamys* and other Velloziaceae hence its inclusion into the family (Salatino et al., 2001). From this phylogenetic analysis, shared genes could be as well provide more reliable phylogenetic insights for species that have undergone genome-wide rearrangement and gene losses. The phylogenetic analyses done so far are largely increasing our understanding of the evolutionary relationship among species in Velloziaceae. Although our results clarified the phylogenetic relationships of the seven Velloziaceae together with species of Order Pandanales, more cp genome of the family and Order need to be sequenced and analyzed to completely understand the phylogeny. Low taxon sampling may produce inconsistencies in the topology of the tree (Leebens-Mack et al., 2005).

### Morphological Comparison

Integrating morphology into phylogenetic analyses is important as it reveals suites of phenotypic novelties that characterize molecular classification hence assists systematists come up with species and clades (Lee and Palci, 2015). In this section we review the morphological importance as specifically used in the classification of *A. bracteata* P.C. Kao. Velloziaceae are xeromorphic and sometimes tree-like monocots with persistent leaf-sheath (Stevens, 2002). Despite the discordance in the treatment of the family Velloziaceae, morphological characters have always provided a foundation in the classification of the family. Velloziaceae are xerophytes adapted to inselbergs which generally favors their endemism (Behnke et al., 2013). The taxonomic history of Velloziaceae is linked mainly to its floral characters, stamen and stigma (Mello-Silva et al., 2011), however, this was misleading because of its variations among the taxa. The anatomical characters that were first used to classify *A. bracteata* were very unique among other monocotyledonous plants including the eustele in rhizome, protostele in root and leaf-stem compound structure in scape. Due to this unique structure in the vascular bundles, it was classified in a separate family Acanthochlamydaceae. However, basing on the morphological and molecular phylogenetic studies, even in this present study molecular analysis, shows a close relationship to the Velloziaceae. This in turn brought into lime-light the close relationship between the Handgun mountains and the African tropical regions, and its classification into family Velloziaceae. *Acanthochlamys* is clearly sister to but morphologically and anatomically different from the rest of the family, with exception of its sieve tube plastids which seem rather similar. The family Velloziaceae is supported currently by at least four-character states: Persistent leaves, presence of abscission zone, two phloem strands and violet tepals (Mello-Silva et al., 2011). In this section, the morphological characters summary among the African genera and the Asian genera are summarized in Table 4 above. Morphological summary of the three species of the family, despite their geographical occurrence differences, fit the classification into similar taxa.

**TABLE 3:** Group of genes encoded in the *Acanthochlamys bracteata, Xerophyta spekei, and X. viscose*.

Function	Categories of the genes	Name of the genes
Self-replication	transfer RNAs	trnA-UGC *, *trnC-GCA*, *trnD-GUC*, *trnE-UUC*, *trnF-GAA*, *trnM-CAU*, *trnG-GCC*, *trnG-UCC*, trnI-CAU *, trnI-GAU *, *trnK-UUU*, trnL-CAA *, *trnL-UAA*, *trnL-UAG*, trnH-GUG*, trnN-GUU *, *trnP-UGG*, *trnQ-UUG*,trnR-ACG *, *trnR-UCU*, *trnS-GCU*, *trnS-GGA*, *trnS-UGA*, *trnT-GGU*, *trnT-UGU*, trnV-GAC *, *trnV-UAC*, *trnW-CCA*, *trnY-GUA,trnK-UUU.*
	ribosomal RNAs	rrn4.5 *, rrna5 *, rrn16 *, rrn23 *
	RNA polymerase	*rpoA*, *rpoB*, *rpoC1*, *rpoC2*
	Small subunit of ribosomal proteins (SSU)	*rps2*, *rps3*, *rps4*, rps7 *, *rps8*, *rps11*, rps12 *, *rps14*, *rps15*, *rps18*, rps19 *
	Large subunit of ribosomal proteins (LSU)	rpl2 *, *rpl14*, *rpl16*, *rpl20*, *rpl22*, rpl23 *, *rpl32*, *rpl33*, *rpl36*
Genes for photosynthesis	Subunits of NADH-dehydrogenase	*ndhA*, ndhB *, *ndhC*, *ndhD*, *ndhE*, *ndhF*, *ndhG*, *ndhH*, *ndhI*, *ndhJ*, *ndhK*
	Subunits of photosystem I	*psaA*, *psaB*, *psaC*, *psaI*, *psaJ*
	Subunits of photosystem II	*psbA*, *psbB*, *psbC*, *psbD*, *psbE*, *psbF*, *psbH*, *psbI*, *psbJ*, *psbK*, *psbL*, *psbM*, *psbN*, *psbT*, *psbZ*
	Subunits of cytochrome b/f complex	*petA*, *petB*, *petD*, *petG*, *petL*, *petN*
	Subunits of ATP synthase	*atpA*, *atpB*, *atpE*, *atpF*, *atpH*, *atpI*
	Large subunit of rubisco	*rbcL*
Other genes	Translational initiation factor	*infA*
	Protease	*clpP*
	Maturase	*matK*
	Subunit of Acetyl-CoA-carboxylase	*accD*
	Envelope membrane protein	*cemA*
	C-type cytochrome synthesis gene	*ccsA*
Genes of unknown function	hypothetical chloroplast reading frames (*ycf*)	ycf1 *, ycf2 *, *ycf3*, *ycf4*

*Duplicated genes.

**TABLE 4:** Comparison of morphological characters between *Xerophyta* and *Acanthochlamys*.

	Character	*Xerophyta*	*Acanthochlamys*
**Anatomy**			
	**Leaf**	Leaf flat and V-shaped with a median adaxial groove; deciduous or rarely persistent	Leaves suberect, grooved on both surfaces
	**Habit**	Xerophytic Small to large perennial herbs or Shrubs	Xerophytic dwarf herb
	**Flowers**	Flowers Large (ca.4.5–7(-12) cm, Bisexual, actinomorphic	Flowers small (Ca. 1cm long), Bisexual, actinomorphic; pedicel very short; flowers mostly pink
	**Placentation**	Axile	Parietal placenta in upper part of the ovary and axile placenta at lower part
**Perianth**	Perianth above the Ovary, white, blue, mauve, or yellow in color	Perianth corona-like, red to purple
	**Filaments**	Filaments flattened, adnate to the tepals	Filaments merges tepals, nearly absent
	**Stigma**	Stigmas linear, erect.	stigma (2 or)3-lobed
	**Root**	Tufted roots	Rhizomes short, with tufted roots
	**Stamen**	Stamen 6	Stamen 6 or numerous
	**Ovary**	Inferior	Inferior
	**Fruit**	Capsule	Capsule, obliquely lanceolate, slightly 3-angled, beaked
	**Nucellus**	Axile placenta, placentae lobed spreading	Tenuinucellate, no nucellar
**Embryology**	**Embryo sac**	Polygonum type	Polygonum or Allium type
	**Endosperm**	Helobial	Nuclear
**Chromosome Number**		*n* = 24	2*n* = 38, *n* = 19
**Geographic Distribution**		Africa, Madagascar, Arabian Peninsula	W Sichuan and SE Tibet

## Conclusion

The complete chloroplast genome of the *A. bracteata* was reported and comparative and phylogenetic analyses with the two species from genus *Xerophyta* revealed similarities in their genomic structure and composition. Additionally, it provided valuable genetic information for further studies on the three species, *A. bracteata*, *X. spekei*, and *X. viscosa*, in terms of chloroplast sequence variations, assembly and evolution. Valuable genetic resources such as SSRs, large repeats and variable loci can be used as genetic markers important for barcoding. Additionally, to understand the sequence divergences in terms of phylogeny, the genetic markers can perhaps be used in the phylogenetic tree analysis upon further analyses. Furthermore, since these species are desiccation and drought tolerant, the genetic markers can be used in the agricultural sector with broad studies on the species compatibility in breeding research.

## Genome Sequence Data

The complete chloroplast sequences *Acanthochlamys bracteata* and other species used in the study are available in GenBank, https://www.ncbi.nlm.nih.gov/(Supplementary Table 6).

## Data Availability Statement

The datasets presented in this study can be found in online repositories. The names of the repository/repositories and accession number(s) can be found in the article/Supplementary Material.

## Author Contributions

VW, XD, G-WH, Q-FW, and RG participated in design of the study can carried out the experiment. XD and G-WH collected the materials. VW, MO, EM, GO, MG, PK, and J-XY contributed in data analysis and draft manuscript writing. VW, XD, G-WH, MG, MO, and EM revised the draft manuscript. All the authors read and approved the final version of the manuscript.

## Conflict of Interest

The authors declare that the research was conducted in the absence of any commercial or financial relationships that could be construed as a potential conflict of interest.

## Footnotes

1 https://chlorobox.mpimp-golm.mpg.de/geseq.Html

2 http://pgrc.ipk-gatersleben.de/misa/misa.html

3 https://www.megasoftware.net
