# Supplementary material for: Complete Chloroplast Genomes of Acanthochlamys bracteata (China) and Xerophyta (Africa) (Velloziaceae): Comparative Genomics and Phylogenomic Placement
Source: Front Plant Sci. 2021 Jun 14;12:691833. doi: 10.3389/fpls.2021.691833 (PMC8238049; doi:10.3389/fpls.2021.691833)
Supplement: Supplementary file 1 [file Data_Sheet_1.zip › Table S1 The genes with introns and exons in the U. rockii chloroplast genome and their locations.docx]

**Table S1 The genes with introns and exons in the *A.bracteata* chloroplast genome and their locations.**

| **Gene** | **Location** | **Exon I (bp)** | **Intron I (bp)** | **Exon II (bp)** | **Intron II (bp)** | **Exon III (bp)** |
| --- | --- | --- | --- | --- | --- | --- |
| *rpl16* | LSC | 401 | 958 | 8 |  |  |
| *atpF* | LSC | 410 | 796 | 143 |  |  |
| *rpoC1* | LSC | 1616 | 696 | 431 |  |  |
| *ycf3* | LSC | 158 | 798 | 227 | 721 | 125 |
| *clpP* | LSC | 251 | 660 | 290 | 814 | 68 |
| *rpl2* | IRb | 431 | 663 | 392 |  |  |
| *ndhB* | IRa | 755 | 707 | 776 |  |  |
| *petB* | LSC | 5 | 817 | 641 |  |  |
| *petD* | LSC | 5 | 778 | 515 |  |  |
| *ndhA* | SSC | 550 | 849 | 540 |  |  |
