# Supplementary material for: Complete Chloroplast Genomes of Acanthochlamys bracteata (China) and Xerophyta (Africa) (Velloziaceae): Comparative Genomics and Phylogenomic Placement
Source: Front Plant Sci. 2021 Jun 14;12:691833. doi: 10.3389/fpls.2021.691833 (PMC8238049; doi:10.3389/fpls.2021.691833)
Supplement: Supplementary file 1 [file Data_Sheet_1.zip › Table S4 Positively selected sites detected.docx]

|  |  |  |  | **M8** |
| --- | --- | --- | --- | --- |
|  |  |  | |  |
|  | **Gene Name** | **Selected Sites 1** | | **Selected Sites 2** |
|  |  |  |  |  |
|  | ***rps16*** | **0.994**** |  | **0.996**** |
|  | ***atpI*** | **0.996**** |  |  |
|  | ***atpF*** |  |  | **0.991**** |
|  | ***atpH*** |  |  | **0.998**** |
|  | ***rpoC2*** | **0.996**** | | **0.998**** |
|  | ***psbZ*** | **0.995**** | |  |
|  | ***rps14*** | **0.991**** | |  |
|  | ***ndhC*** | **0.996**** | |  |
|  | ***rbcL*** | **0.995**** | | **0.969**** |
|  | ***accD*** | **0.996**** | | **0.998**** |
|  | ***psaA*** |  | | **0.993**** |
|  | ***atpB*** |  | | **0.992**** |
|  |  |  | |  |
|  |  | ***p<0.05; **p< 0.01.** | |  |

**Table S4**. **Positively selected sites detected in the chloroplast genome of Order Pandanales based on Bayes Empirical Bayes (BEB) (site 2) and NEB (site 1) methods.**
