# Supplementary material for: Complete Chloroplast Genomes of Acanthochlamys bracteata (China) and Xerophyta (Africa) (Velloziaceae): Comparative Genomics and Phylogenomic Placement
Source: Front Plant Sci. 2021 Jun 14;12:691833. doi: 10.3389/fpls.2021.691833 (PMC8238049; doi:10.3389/fpls.2021.691833)
Supplement: Supplementary file 1 [file Data_Sheet_1.zip › Table S6 cp genome sequences of from GenBank used in this study.docx]

**Table S6 cp genome sequences of from GenBank used in this study.**

| **Species** | **GenBank accession numbers** |
| --- | --- |
| *Lilium pardanthium* | MG704135 |
| *Lilium gongshanense* | NC_052787 |
| *Fritillaria delavayi* | MN480806 |
| *Fritillaria unibracteata* | NC_044629 |
| *Lilium meleagrinum* | NC_052788 |
| *Lilium sulphureum* | NC_052792 |
| *Lilium regale* | NC_052790 |
| *Lilium sargentiae* | NC_052791 |
| *Lilium nepalense* | NC_052789 |
| *Lilium cernuum* | NC_034840 |
| *Lilium brownii* | NC_035588 |
| *Lilium longiflorum* | KC968977 |
| *Pandanus tectorius* | NC_042747 |
| *Carludovica palmata* | NC_026786 |
| *Xerophyta viscosa* | NC_043880 |
| *Xerophyta spekei* | MN663122 |
| *Croomia heterosepala* | NC_039673 |
| *Croomia japonica* | NC_039672 |
| *Croomia pauciflora* | NC_039674 |
| *Stemona japonica* | NC_039675 |
| *Stemona tuberosa* | MW246829 |
| *Stemona mairei* | NC_039850 |
| *Dioscorea baya* | NC_039854 |
| *Dioscorea sagittifolia* | NC_039854 |
| *Dioscorea abyssinica* | NC_039834 |
| *Dioscorea alata* | NC_039707 |
| *Dioscorea togoensis* | NC_039856 |
| *Dioscorea aspersa* | NC_039807 |
| *Dioscorea paehensilis* | NC_039837 |
| *Dioscorea cayenensis subsp rotundata* | NC_024170 |
| *Dioscorea cayenensis* | NC_039836 |
| *Dioscorea hirtifolia* | NC_039851 |
| *Dioscorea persimilis* | MN585218 |
| *Dioscorea schimperiana* | NC_039855 |
| *Dioscorea japonica* | MT920319 |
| *Dioscorea polystachya* | NC_039855 |
| *Dioscorea nipponica* | MT906794 |
| *Dioscorea quartiniana* | NC_039853 |
| *Dioscorea dumetorum* | NC_039691 |
| *Dioscorea preusii* | NC_039852 |
| *Dioscorea sansibarensis* | NC_039838 |
| *Dioscorea esculenta* | NC_052854 |
| *Dioscorea elephantipes* | NC_009601 |
| *Dioscorea bulbifera* | NC_039708 |
| *Dioscorea villosa* | NC_034686 |
| *Dioscorea collettii* | NC_037717 |
| *Dioscorea quinquelobata* | MT936876 |
| *Dioscorea zingiberensis* | NC_027090 |
| *Dioscorea futschanensis* | NC_039808 |
| *Elaeis guineensis* | NC_017602 |
